# Developing Computerized Adaptive Testing for a National Health Professionals Exam: An Attempt from Psychometric Simulations

**DOI:** 10.5334/pme.855

**Published:** 2023-10-31

**Authors:** Lingling Xu, Zhehan Jiang, Yuting Han, Haiying Liang, Jinying Ouyang

**Affiliations:** 1 Peking University, Beijing, China; 2 School of Psychology, Beijing Language and Culture University, Beijing, China; 3 Institute of Education, University College London, London, United Kingdom

## Abstract

**Introduction:**

The accurate assessment of health professionals’ competence is critical for ensuring public health safety and quality of care. Computerized Adaptive Testing (CAT) based on the Item Response Theory (IRT) has the potential to improve measurement accuracy and reduce respondent burden. In this study, we conducted psychometric simulations to develop a CAT for evaluating the candidates’ competence of health professionals.

**Methods:**

The initial CAT item bank was sourced from the Standardized Competence Test for Clinical Medicine Undergraduates (SCTCMU), a nationwide summative test in China, consisting of 300 multiple-choice items. We randomly selected response data from 2000 Chinese clinical medicine undergraduates for analysis. Two types of analyses were performed: first, evaluating the psychometric properties of all items to meet the requirements of CAT; and second, conducting multiple CAT simulations using both simulated and real response data.

**Results:**

The final CAT item bank consisted of 121 items, for which item parameters were calculated using a two-parameter logistic model (2PLM). The CAT simulations, based on both simulated and real data, revealed sufficient marginal reliability (coefficient of marginal reliability above 0.750) and criterion-related validity (Pearson’s correlations between CAT scores and aggregate scores of the SCTCMU exceeding 0.850).

**Discussion:**

In national-level medical education assessment, there is an increasing need for concise yet valid evaluations of candidates’ competence of health professionals. The CAT developed in this study demonstrated satisfactory reliability and validity, offering a more efficient assessment of candidates’ competence of health professionals. The psychometric properties of the CAT could lead to shorter test durations, reduced information loss, and a decreased testing burden for participants.

## Introduction

The health professions bear a significant responsibility to ensure that all candidates aspiring to enter legal practice demonstrate the necessary level of competence before obtaining licensure. Valid assessment of the competence of health professionals is essential for safeguarding public health, safety, and welfare. While programs of assessment in the education of health professionals often include multiple assessment methods, one of the most frequently used methods is standardized tests, such as multiple-choice item examinations [1,2]. This approach is prevalent in medical education and is commonly seen in national-level examinations, such as the United States Medical Licensing Exam (USMLE) and the Medical Council of Canada Qualifying Exam (MCCQE). Standardized tests primarily using multiple-choice items hold a pervasive presence throughout the domain and are tailored to objectively gauge performance. Moreover, given the time and cost constraints in the medical field, there is a fundamental need for precise, cost-effective assessments of the competence of health professionals. Developing efficient and effective standardized measurement tools that are both brief and reliable is crucial for enhancing stakeholder outcomes and reducing costs. Consequently, there is a critical need for investment in research that generates valid, efficient, and reliable measurement assessments in medical education.

Widely seen in medical education assessment, one of the more commonly applied approaches in standardized tests is that of Classical Test Theory (CTT), which results in summed scores and requires respondents to complete the entire test for comparability, leading to greater burden and potentially lower response quality [3]. Given the time constraints faced by stakeholders in medical education, assessments need to be both concise and reliable for all candidates to decrease costs [4]. This approach aids in extending the utilization of assessment tools to non-specialty healthcare [5]. As such, there is a need for efficient and high-quality assessment tools in standardized tests to facilitate individualized assessment planning. Computerized Adaptive Testing (CAT) has emerged as a promising methodology in this context.

The utilization of CAT methodology has received considerable attention in medical education because of its potential to provide high-quality evaluations quickly [6,7,8]. CAT is a computerized assessment method that dynamically selects items corresponding to an examinee’s ability on the fly from an item bank (i.e., sets of items that measure the construct of interest) and then updates the estimate of the examinee’s ability according to the response to this item. This process is repeated until the examinee’s ability is accurately estimated (i.e., the estimate stays stable even given more items) [9]. Unlike traditional linear standardized tests, CAT furnishes the flexibility to tailor tests to each person through an algorithm that presents items one-by-one, selecting the most informative ones. Consequently, fewer items are presented, reducing candidates’ burden [10].

Modern test theory, also known as Item Response Theory (IRT), underpins the CAT methodology, suggesting that responses to test items are influenced by a latent construct and item characteristics. Within this framework, IRT enables the evaluation of measurement precision at distinct levels of the measured construct. Thus, IRT methods can identify the range of the latent trait continuum for which the item can best discriminate among individuals, and reveal how well different items discriminate at specific levels of the construct. This information can be used to select the most informative (discriminative) items and administer only those to the participant [11]. When the responder submits their answer, the CAT algorithm utilizes IRT principles to estimate the provisional latent construct score and choose the most informative item from the entire set to present subsequently [4,12].

Recent research in medical education assessment has exhibited growing interest in the application of IRT and CAT for standardized tests [8,13]. However, several gaps still exist that warrant further investigation. First, many of the findings stem from specific CAT implementations with restricted item banks, which limits their generalizability to all health professionals’ exams. Second, from a methodological perspective, a wide range of models can be employed within the IRT framework to fit CAT. Yet, no studies on health professionals’ exams have compared different IRT models for their CAT or selected an optimal model based on a test-level model-fit check. Third, it is important to note that there exist a multitude of item selection methods and stopping rules for CAT applications. However, previous studies have not sufficiently compared the performance of these diverse item selection methods and stopping rules against each other. Fourth, the samples used in existing CAT studies for health professionals’ exams come from various countries, with Mercer et al. [6] utilizing an American sample, Sibbald [7] employing a Canadian sample, and Seo and Choi [8] drawing upon a Korean sample. However, no research has developed CAT for health professionals’ exams using a Chinese sample. Therefore, this study aims to address these gaps by developing a novel IRT-based CAT simulation technique to assess the competence of candidates for participating a national health professionals in China, one of the largest healthcare systems in the world. The development of CAT version holds the potential to enhance evidence-based assessment practices for measuring the competence of health professionals and to enable the generation of individually tailored tests for candidates in health professions.

In alignment with previous research, this study will adopt a two-phase methodology to fulfill its objectives. The first phase will involve the creation of a calibrated item bank, ensuring robust psychometric properties, through the application of IRT. Subsequently, in the second phase, the study will evaluate the reliability and validity of the CAT by utilizing both simulated and actual response data.

## Methods

### Overall framework of Developing CAT

**Figure 1:**
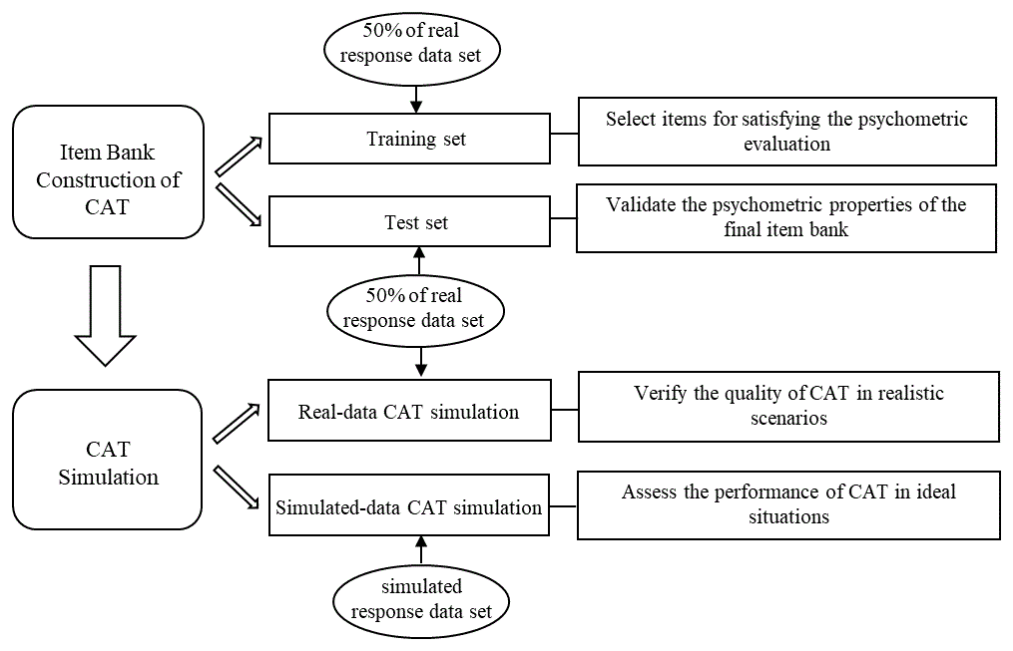
Overall framework of developing CAT.

According to common practice in previous studies [14,15], two steps of analyses were conducted to develop CAT via IRT (see Figure 1). The first analysis was to perform the psychometric evaluation of the item bank for CAT (i.e., Construction of Item Bank CAT), and the second was to simulate the CAT using simulated and real data (i.e., CAT Simulation). We describe these two steps of analyses as follows.

As highlighted by Magis and Raîche [16], any CAT process mandates a calibrated item bank. For a stable psychometric evaluation of the CAT item bank in the initial analysis, real responses collected beforehand were randomly split into a training set (comprising 50% of the real responses) and a test set (containing the remaining 50%). This approach aligns with that of Flens et al. [14]. Specifically, the training set was employed for item selection to meet the psychometric criteria, while the test set was utilized to validate the psychometric properties of the final CAT item bank.

Following the methodology in related works [17,18], CAT simulations were executed with both simulated and real data upon the finalization of the CAT item bank. Specifically, simulated-data CAT simulations were conducted to assess the performance of CAT under ideal conditions. Simulated data were generated based on item parameters and simulated examinee ability. Drawing from existing literature [19], 71 ability levels ranging from -3.5 to +3.5 at intervals of 0.1 were employed, and each level was replicated 100 times to simulate a total of 7100 new examinees. This ability range covered nearly all examinee ability levels (99.96%), with 71 levels and 100 replications each ensuring coverage of a wide array of representative ability points for diverse examinee groups. In contrast, real-data CAT simulations were undertaken to ascertain the quality of CAT in practical contexts. It is important to note that overfitting and overly optimistic results may arise when the same sample is used for both item parameter estimation and CAT simulation [20]; thus, real data from the test set utilized for CAT item bank construction were applied in the real-data CAT simulations.

### Examinees and Initial Item Pool

In the initial stage of this study, items were sourced from the Standardized Competence Test for Clinical Medicine Undergraduates (SCTCMU), launched by National Medical Examination Center and National Center for Health Professions Education Development in China. The SCTCMU was delivered nationwide and used to assess students’ learning outcomes in basic and clinical medical sciences at the end of the 4th-year of their 5-year undergraduate studies in clinical medicine. The SCTCMU includes two steps, the first step is clinical skill in the form of objective structured clinical examination and the second one is medical knowledge delivered as computerized multiple-choice items. It is based on the content specified in the National Chinese Medical Qualification Examination Standards to determine the scope of content; Corresponding proportions in each subject regarding medical knowledge are shown in Table S1 of Appendix B. The medical knowledge section includes 300 multiple-choice items, each scored dichotomously and featuring four distractors and one correct answer.

For the purposes of this study, the initial CAT item bank comprised 300 dichotomously scored multiple-choice items from the medical knowledge portion of the SCTCMU. In accordance with prior research [21], a sample size of at least 1000 is considered enough for acceptable item parameter estimates for unidimensional IRT models through simulation studies in the item bank calibration. Consequently, a cohort of 2000 Chinese medical students (who enrolled in the fall semester of 2018) was randomly chosen from the full set of SCTCMU examinees. Specifically, the real responses of 1000 students were utilized to create the CAT item bank, while the real responses of the remaining 1000 students were employed to simulate CAT for the purpose of verifying the precision and validity of the algorithm. This study, involving human participants, received approval from the Biomedical Ethics Committee of Peking University (IRB00001052-22070).

### Construction of CAT Item Bank

The construction of the CAT item bank in this study involved a psychometric evaluation conducted via IRT, utilizing real data from the training set. We specifically assessed five assumptions: unidimensionality, model selection, local independence, item fit, and item discrimination. These assumptions that demand statistical testing are foundations to CAT’s construction; their conceptual explanations, rules of thumbs, and relevant information are documented in Appendix A such that readers caring less statistical/programming details can skip the part. That said, only by passing these assumption tests should CAT be allowed to initiate:

1. satisfying unidimensionality [22];
2. exhibiting adequate fit to the IRT model [14,15];
3. meeting the local independence assumption in IRT [23];
4. showing satisfactory item fit [20];
5. displaying acceptable item discrimination estimates exceeding 0.50 [24].

Upon passing the tests of these assumptions, items that met the psychometric criteria were retained for the construction of the final CAT item bank. Furthermore, to test the performance of this method on the test set after iterating all item reduction methods on the training set, a similar evaluation (including unidimensionality, model selection, local independence, item fit, and item discrimination) was conducted on the test set. The psychometric evaluation related to the CAT item bank construction was performed using Mplus 7.0 [25] and the R package “mirt” [26].

### CAT Simulation

**Figure 2:**
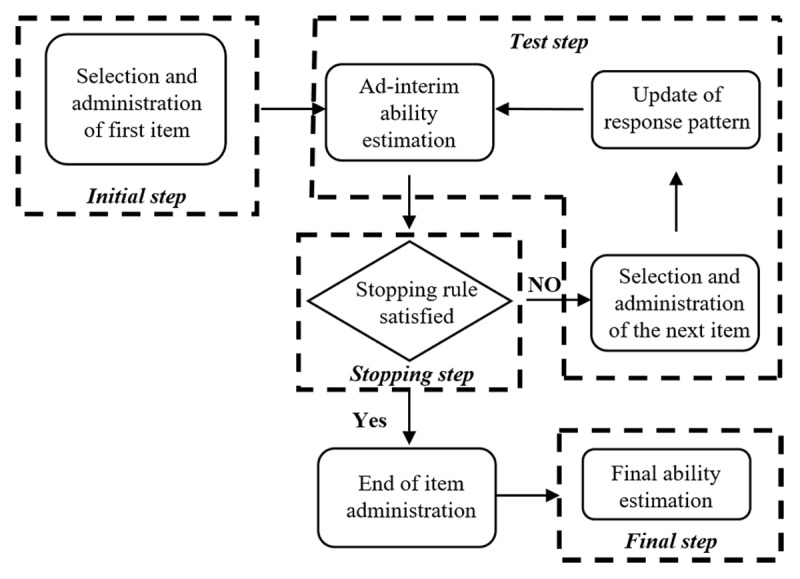
Schematic illustration of CAT process.

After constructing the final CAT item bank, we conducted CAT simulations using both simulated and real data. CAT process can be schematically decomposed in four successive steps (see Figure 2).

1. The *initial step*, or starting step, involves selecting a single item to initiate the CAT process.
2. The second step is the *test step*, in which the items are iteratively and optimally selected and administered, and the ability level estimate is re-computed after each item administration. Administration means the action that a computer selects and delivers items to examinees. Specifically, after administering the starting item, the ability value is estimated using the expected a posteriori method (EAP) [19], based on the item response and item parameters. The EAP algorithm was chosen for estimating examinees’ ability due to its efficiency and stability [19]. Subsequently, the next item to be administered is selected from the eligible items according to the maximum Fisher information (MFI) criterion [27], a widely-used item selection criterion in adaptive testing. The MFI criterion stipulates that among the set of eligible items, the item with the highest information at the current estimated ability level of the examinee should be selected next. In addition to this, non-statistical constraints, such as content constraints, are incorporated into item selection by employing the maximum priority index (MPI) [28] approach. Under this method, the item with the largest priority index, rather than the largest Fisher information, is chosen as the next item. Following this, the examinee’s response is recorded, and the response pattern is updated.
3. The third step is the *stopping step*, which defines the rules to make the adaptive item administration stop. In this study, various fixed-length stopping rules were applied, including terminating the test after 20, 40, 60, or 121 items were applied to find a better one. To investigate the rules between different precisions, CAT was conducted under specified standard errors (SEs) of theta (SEs of 0.500, 0.447, 0.387, and 0.316). These SEs correspond to measurement reliabilities of 0.750, 0.800, 0.850, and 0.900, respectively, as determined by the conversion formula between reliability and SE in IRT.
4. The *final step* yields the final ability estimation, and the number of items to which the examinee responded is also documented. All CAT simulations were conducted using the R package “catR” [29]. The evaluation criteria encompassing characteristics, reliability, content validity, and criterion-related validity are outlined in Table S3 of Appendix B.

## Results

### Construction of CAT Item Bank

Following the psychometric evaluation outlined previously, we generated a final CAT item bank consisting of 121 items. These items satisfied the assumptions of unidimensionality and local independence within the context of IRT, exhibited a good fit to the two-parameter logistic model (2PLM) [30], demonstrated satisfactory item fit, and had high discriminative item parameters (see Figure S2 in Appendix B). Subsequent validation with the test set affirmed that the items included in the final CAT item bank possessed suitable item characteristics. Details regarding the development process of the item bank for the CAT can be found in Appendix B.

### CAT Simulation

#### Results Based on the Simulated-data CAT Simulation

##### Characteristics

Tables 1 and 2 present the results of the simulated-data CAT simulation using the MFI and MPI methods under seven stopping rules based on Length (item) = 20/40/60 and *SE* (theta) = 0.500/0.447/0.387/0.316. For the simulated-data CAT simulation utilizing the MFI method, the average number of selected items ranged from 19.483 to 62.370, which corresponded to 16.1% to 51.5% of the original full-set items being employed across the seven stopping rules. The mean SE of theta (i.e., ability) for the different stopping rules varied from 0.316 to 0.488, reflecting the respective measurement precision achieved with each rule. Notably, the outcomes of CAT simulations using both the MFI and MPI methods exhibited consistent performance. Furthermore, the number of items selected in CAT simulations employing the MPI method under stopping rules based on *SE* (theta) = 0.500/0.447/0.387/0.316 was even fewer than in simulations using the MFI method.

**Table 1:** CAT simulation statistics using MFI method under several stopping rules.


CAT SIMULATION	STOPPING RULES	NUMBER OF ITEMS		MEAN OF SE (THETA)	MARGINAL RELIABILITY	*V̅*	BONFERRONI-ADJUSTED *p*-VALUES

		MEAN	% ALL				

Simulated-data CAT simulation	None	121	100	0.277	0.916	0.000	1.000

	Length (item) = 60	20	16.5	0.431	0.804	1.226	0.911

	Length (item) = 40	40	33.1	0.351	0.868	1.312	0.953

	Length (item) = 20	60	49.6	0.316	0.892	0.810	0.900

	*SE* (theta) ≤ 0.316	62.370	51.5	0.337	0.884	1.233	0.168

	*SE* (theta) ≤ 0.386	41.494	34.3	0.390	0.847	1.871	0.055

	*SE* (theta) ≤ 0.447	28.013	23.2	0.441	0.805	1.632	0.051

	*SE* (theta) ≤ 0.500	19.483	16.1	0.488	0.761	1.876	0.052

Real-data CAT simulation	None	121	100	0.227	0.946	0.000	1.000

	Length (item) = 60	20	16.5	0.390	0.844	1.518	0.928

	Length (item) = 40	40	33.1	0.307	0.903	1.810	0.960

	Length (item) = 20	60	49.6	0.269	0.925	0.759	0.917

	*SE* (theta) ≤ 0.316	41.579	34.4	0.316	0.900	2.577	0.303

	*SE* (theta) ≤ 0.386	24.329	20.1	0.381	0.854	2.233	0.354

	*SE* (theta) ≤ 0.447	16.527	13.7	0.438	0.808	2.515	0.716

	*SE* (theta) ≤ 0.500	12.674	10.5	0.487	0.762	2.814	0.284


*Note*: All items in the item bank were selected; theta, ability; % all, the percentage of the mean numbers of administered items in the full-item bank; *V̅* is a test nonstatistical constraint violation.

**Table 2:** CAT simulation statistics using MPI method under several stopping rules.


CAT SIMULATION	STOPPING RULES	NUMBER OF ITEMS		MEAN OF SE (THETA)	MARGINAL RELIABILITY	*V̅*	BONFERRONI-ADJUSTED *p*-VALUES

		MEAN	% ALL				

Simulated-data CAT simulation	None	121	100	0.277	0.916	0.000	1.009

	Length (item) = 60	20	16.5	0.453	0.785	1.000	0.928

	Length (item) = 40	40	33.1	0.355	0.865	1.059	0.948

	Length (item) = 20	60	49.6	0.318	0.891	1.270	0.602

	*SE* (theta) ≤ 0.316	40.652	33.6	0.308	0.905	2.414	0.328

	*SE* (theta) ≤ 0.386	25.021	20.7	0.372	0.861	1.990	0.248

	*SE* (theta) ≤ 0.447	17.427	14.4	0.429	0.816	2.754	0.120

	*SE* (theta) ≤ 0.500	12.997	10.7	0.477	0.772	2.401	0.334

Real-data CAT simulation	None	121	100	0.227	0.946	0.000	1.000

	Length (item) = 60	20	16.5	0.412	0.826	1.000	0.904

	Length (item) = 40	40	33.1	0.312	0.900	1.233	0.104

	Length (item) = 20	60	49.6	0.273	0.923	2.032	0.055

	*SE* (theta) ≤ 0.316	33.031	27.3	0.313	0.902	1.044	0.308

	*SE* (theta) ≤ 0.386	19.802	16.4	0.380	0.855	0.354	0.443

	*SE* (theta) ≤ 0.447	14.121	11.7	0.437	0.809	1.972	0.451

	*SE* (theta) ≤ 0.500	10.964	9.1	0.486	0.764	2.281	0.473


*Note*: All items in the item bank were selected; theta, ability; % all, the percentage of the mean numbers of administered items in the full-item bank; *V̅* is a test nonstatistical constraint violation.

##### Reliability and Validity

As shown in Tables 1 and 2, marginal reliabilities (i.e., the average reliability for all examinees) under the various stopping rules spanned from 0.761 to 0.892. Our analysis confirmed that the final item bank had good content validity, as detailed below: (1) the content coverage of the final item bank remains consistent with that of the initial item bank, both containing 16 specific content areas that met the requirements of the test blueprints (see Figure S1 and Table S5 in Appendix B). (2) By comparing the content distribution of items in the initial item bank over the past three years, we identified a consistent pattern (see Table S2 in Appendix B). This indicates that the content distribution of the initial item bank used in this study is reasonable. Our study also found that the content distribution of the final item bank exhibited similarities to that of the initial item bank (see Figure S1 of Appendix B). (3) This study indicated successful constraint management, as evidenced by the extremely small values of non-statistical constraint violations in the tests and the fact that there was no statistically significant difference in content distribution (i.e., Bonferroni-adjusted *p*-values higher than 0.050) between the test blueprints and the tests assembled for the participants. Furthermore, Pearson’s correlations between the estimated ability of CAT and the aggregate scores of the SCTCMU were generally high (ranging from 0.864 to 0.939) and statistically significant at the 0.010 level (two-tailed) under all seven stopping rules, indicating high criterion-related validity of CAT. Overall, these results suggest that simulated-data CAT simulation achieved sufficient reliability and validity.

#### Results Based on the Real-data CAT Simulation

Tables 1 and 2 also display the outcomes of real-data CAT simulation using the MFI and MPI methods under the seven stopping rules based on Length (item) = 20/40/60 and *SE* (theta) = 0.500/0.447/0.387/0.316. The performance of the real-data and simulated-data CAT simulations were consistent. Moreover, the number of items required in the real-data CAT simulation was even lower than in the simulated-data CAT simulation. Taken together, these findings demonstrate that CAT performed well in the real-data simulation.

## Discussion

In this study, we used a novel IRT-based CAT simulation technique to develop a computer-adaptive version for the assessment of candidates’ competence of health professionals within the Chinese population. The findings reveal the successful implementation of CAT in reducing the test length during administration. This is, which could advance evidence-based assessment practices and facilitate the creation of individually customized tests for candidates in health professions.

In the present study, seven stopping rules and two item selection methods were employed to conduct simulated CATs, yielding several notable findings: (1) As expected, a higher measurement precision required more items to be administered on average. Conversely, when the fixed test length was reduced, measurement accuracy decreased. In addition, a positive correlation was observed between latent competence estimates generated by full and adaptive assessments as measurement requirements increased. As the required measurement precision increased, the marginal reliability of latent competence estimates was less affected by measurement error. Notably, even with relaxed measurement precision requirements, the information loss was surprisingly minimal. For example, CAT estimates using the stopping rule *SE* (theta) ≤ 0.316, administering one half to one-third of the items per respondent, showed high correlations (ranging from 0.872 to 0.921) with the full assessment score. Similarly, CAT estimates using the stopping rule *SE* (theta) ≤ 0.500, administering about one-sixth to one-tenth of the items per respondent, also exhibited strong correlations (ranging from 0.871 to 0.939) with the original score. (2) In general, CAT simulations employing the MPI method had a lower average number of administered items than those utilizing the MFI method across stopping rules of *SE* (theta) = 0.500/0.447/0.387/0.316. This discrepancy could potentially be attributed to the weight assigned to non-statistical constraints within the MPI method. Future research could explore the effects of adjusting the weight of non-statistical constraints within the MPI method to determine the average number of items administered in CAT applications.

When applying CAT in practice or research, different results may be produced by simulated and real CAT administration [26] in that there are many factors that affect individuals’ responses in a real situation, such as answering time, individual mood, test environment, etc. Fortunately, our findings are consistent with the results of a previous study [27], indicating that the results of simulated CAT align with actual CAT. The current study demonstrated that CAT had acceptable reliability and validity. Additionally, our analysis was conducted sequentially, following an order specified by IRT for evaluating item banks (i.e., unidimensionality, model-fit, local independence, item-fit, item discrimination). It is plausible that the order for evaluating different item/test properties influenced the final item bank. A more appropriate approach needs to be explored in future studies, such as mapping the quality of all items at each step and making decisions regarding item retention based on this information.

The implications of this research are considerable in a clinical context. On the one hand, CAT offers the ability to dynamically adapt item difficulty to the ability level of the candidate, thus enabling a more precise assessment of the competence of candidates for health professionals. This approach facilitates the identification of health professionals with the requisite abilities, ultimately enhancing the quality and safety of healthcare services. On the other hand, the implementation of standardized tests using the CAT methodology would reduce the time, cost, and human effort required to assess the competence of candidates for health professionals, as it limits the number of items administered. This also reduces the bulkiness of the process and in turn the negative feelings associated with it.

The present study has highlighted the potential for enhancing the efficiency of medical education assessment, several issues still require attention. First, the SCTCMU was selected to determine the criterion-related validity of CAT, but it does not show the relationship between CAT and other external criteria; that said, obtaining other related scales or tests could be used to support criterion-related validity. Second, the final item bank of CAT was derived from the real items of SCTCMU, and the initial item for each examinee was randomly selected from this real item bank. In subsequent research, dummy items could be considered for use in simulations as initial items for examinees. This could help avoid imprecise estimations of examinee abilities due to nervousness. Third, we employed the 2PLM-based MFI and MPI criterions to select subsequent items for each examinee in the real-data CAT simulation. This study estimated examinee ability parameters based on their real response data, as we had pre-acquired real response data for each examinee in the full-length test. To maintain a fair comparison between the two CAT simulations, we used the same practice in the CAT simulation of simulated data. Future research could aim to optimize CAT calibration, so that examinees have approximately an 80% chance of answering items correctly, potentially leading to more satisfactory test outcomes.

In the present study, we investigated the use of CAT for evaluating the candidates’ competence of health professionals. The simulation studies included a limited number of items, and the outcomes were only slightly different from those of the full-length tests. Consistent with previous research on CAT [8,13], our findings suggest that CAT offers a valuable approach for enhancing the efficiency of testing procedures while reducing the assessment burden on candidates in health professions. We hope that these results will encourage both medical education assessment researchers and practitioners to apply CAT procedures more often to their full-length tests. Our findings lend support to the feasibility of developing genuine CATs for the assessment of candidates in health professions in the future.

## Additional File

The additional file for this article can be found as follows:

10.5334/pme.855.s1 Appendices. Appendix A and B.
