# Supplementary material for: Developing Computerized Adaptive Testing for a National Health Professionals Exam: An Attempt from Psychometric Simulations
Source: Perspect Med Educ. 2023 Oct 31;12(1):462–71. doi: 10.5334/pme.855 (PMC10624130; doi:10.5334/pme.855)
Supplement: Appendices. — Appendix A and B. [file pme-12-1-855-s1.pdf]

Appendix A

Item Bank Construction of CAT

Unidimensionality

In item response theory (IRT), the assumption of unidimensionality is crucial. An item bank is considered unidimensional when an individual's responses to items are primarily influenced by the underlying trait that the item measures and not from other elements. Evaluating the unidimensionality of an item bank is a necessary step in IRT applications [1]. In this study, both factor analytic and IRT frameworks were employed to assess unidimensionality.

First, the data were randomly partitioned into two equal subsets (n = 500 each), with one set designated for exploratory factor analysis (EFA) and the other for confirmatory factor analysis (CFA). In the EFA, parallel analysis was employed as the criterion to determine the number of meaningful factors. Items with factor loadings greater than 0.300 and statistically significant at p = 0.050 were retained for further development of the item bank. In the CFA, fit indices such as the root mean square error of approximation (RMSEA), comparative fit index (CFI), and Tucker-Lewis index (TLI) were used to assess the model's goodness of fit. According to the literature, acceptable fit is indicated by RMSEA ≤ 0.08, CFI ≥ 0.90, and TLI ≥ 0.90 [2].

Furthermore, unidimensionality was evaluated through a descriptive analysis of the standardized residuals of item responses based on the IRT model. A goodness-of-fit test, derived from the fitted IRT model, was conducted to detect

potential violations of the unidimensionality assumption within the test data [3]. The M_2 statistic was utilized to assess the overall goodness of fit of the parametric model [4].

Test Fit and IRT Model Selection

In the framework of IRT, selecting an appropriate model for parameter estimation is crucial. In this study, three widely-used dichotomous IRT models were considered: the two-parameter logistic model (2PLM) [5], the three-parameter logistic model (3PLM) [6], and the Rasch model [7]. The selection of the optimal IRT model for further analysis was based on the following test-level model fit indices: -2 log-likelihood (-2LL) [8], Akaike's information criterion (AIC) [9], Bayesian information criterion (BIC) [10], and the M_2 statistic [4]. Lower values of these indices indicate a better model fit. Additionally, the goodness-of-fit test (M_2 statistic) was not statistically significant, suggesting that the selected model provided a good fit to the data.

Local Independence

In the framework of IRT, it is important to assess local dependence to ensure that parameter estimates are not unduly influenced by correlated item pairs. In this study, local dependence was evaluated using residual correlations and Yen's Q3 statistic [11]. Following the recommendations of a previous study [12], We considered the presence of local dependence when the residual correlation values were greater than or equal to 0.200, and Yen's Q3 statistic values were 0.360 or higher.

Item Fit

Evaluating item fit is a crucial step in IRT analysis [13]. In this study, the $S\text{-}X^2$ statistic was used to assess item fit [14, 15], which quantifies the discrepancies between observed and expected response frequencies. Items with a *p*-value of $S\text{-}X^2$ less than 0.010 were identified as misfitting and were subsequently removed from the item bank.

Item Discrimination

In IRT, the discrimination parameter is an essential metric for evaluating item quality. Chang and Ying [16] proposed that a discrimination value ranging from 0.500 to 2.500 is considered acceptable for an item parameter. In this study, items with discrimination values below 0.500 were excluded from the item bank to ensure a high-quality CAT item bank.

Appendix B

Results

The development procedure for the CAT item bank is illustrated in Fig. S2, detailing the number of items retained or deleted at each step, along with the reasons for deletion.

Unidimensionality

In the initial item pool, 172 items were excluded based on EFA results. Specifically, these items were removed due to factor loadings less than 0.300 or non-significance ($p > 0.050$). Subsequently, a one-factor model CFA was conducted using the remaining 128 items. Results indicated an acceptable model fit with a RMSEA of 0.023, a CFI of 0.904, and a TLI of 0.910. These findings supported the unidimensionality of the 128-item subset.

Test Fit and IRT Model Selection

Among the considered models, the 2PLM demonstrated the best fit for the remaining items, as evidenced by the smallest values of -2LL, AIC, and BIC (see Tab. S4). Furthermore, the goodness of fit, evaluated by the M_2 statistic (M_2 = 10437.2, df = 7802, p = 0.080), was not statistically significant (see Tab. S4). Therefore, the 2PLM was selected for subsequent analyses of the final CAT item bank.

Local Independence

No items were removed based on local independence criteria, as all items exhibited residual correlations less than 0.200 and absolute Q3 values below 0.360. The remaining items were deemed to satisfy the local independence assumption.

Item Fit

One item was found to be a misfit for the 2PLM, as indicated by a p-value of the $S\text{-}X^2$ statistic below 0.010. Following the removal of this item, the remaining 127 items were re-evaluated, and all were retained as their p-values for the $S\text{-}X^2$ statistic were above 0.010.

Item Discrimination

The item parameters for the CAT item bank were calibrated using the 2PLM. In this process, six items were removed due to discrimination values below 0.500, resulting in a final item bank consisting of 121 items with satisfactory discrimination power.

The above steps produced a 121-item set of CAT (see Tab. S5) that satisfied the unidimensionality and local independence hypotheses in IRT, fitted the 2PLM well,

had good item fit, and high item discrimination parameters. Moreover, responses from a real sample of 1000 subjects were used to validate the psychometric properties of the CAT final item set. A one-factor CFA of the remaining 121-item set revealed acceptable fit indices (RMSEA = 0.020, CFI = 0.915, and TLI = 0.928), confirming the unidimensionality of the final item set. This result is consistent with the assumption of unidimensionality for large-scale exams such as the United States Medical Licensing Examination (USMLE), the Professional and Linguistic Assessments Board (PLAB), and the National Council Licensure Examination for Registered Nurses (NCLEX-RN) [17-19], and with the characterization of unidimensional IRT models [20-23]. Furthermore, the goodness-of-fit test was not statistically significant (M_2 = 10012.5, df = 6936, p = 0.120), indicating that the CAT final item set represented a single construct. Additionally, no items showed local dependence (residual correlations: < 0.200; Q3 values: < 0.360), and all items fit the 2PLM ($S\text{-}\chi^2$ > 0.010). None of the items in the final set had discrimination values below 0.500. These analyses, based on responses from the 1000 subjects, confirmed that the items in the final CAT item bank exhibit acceptable item characteristics.

Table S1 Summary of content and scope of Standardized Competence Test for Clinical Medicine Undergraduates (SCTCMU)

Subject	Content	Key points	Proportion
Preclinical medicine	anatomy, biochemistry, physiology, pathophysiology, microbiology, immunology, pathology, pharmacology	understanding and application of knowledge related to human health and disease	40~45%
Medical humanities	medicopsychology, medical ethics, sanitary legislation	basic knowledge and important principles	5~10%
Preventive medicine	preventive medicine	important concepts, basic principles and applications in disease prevention and control	5~10%

Clinical medicine	internal medicine, surgery, obstetrics and gynecology, pediatrics	symptoms and signs, etiology and pathogenesis, diagnosis and differential diagnosis, principle of treatment	40~45%

Table S2 Content distribution of SCTCMU across different years

Subject	Year 2021		Year 2022		Year 2023	
	Number of items	% all	Number of items	% all	Number of items	% all
Preclinical medicine	125	41.67	128	42.67	128	42.67
Clinical medicine	128	42.67	125	41.67	125	41.67
Medical humanities	24	8.00	24	8.00	24	8.00
Preventive medicine	23	7.67	23	7.67	23	7.67

Table S3 Evaluation criteria

**Characteristics**	**Reliability**	**Content Validity**	**Criterion-related Validity**
1. mean of the selected items	1. mean standard error of theta<br>2. marginal reliability	1. content coverage<br>2. content distribution<br>3. constraint management	1. correlation between the CAT and the SCTCMU

None. SCTCMU = Standardized Competence Test for Clinical Medicine Undergraduates; two indices were used in constraint management analysis: a test nonstatistical constraint violation was to check whether the test sequentially assembled for each participant meets all the specified nonstatistical constraints; a Bonferroni adjusted *p*-value was used to determine whether there is a statistically significant difference in content distribution between the test blueprint and the test assembled for the participants.

Table S4 Model-fit indices

**Model**	**-2LL**	**AIC**	**BIC**	M_2
Rasch Model	135460.1	135718.1	136351.2	11380.3
2PLM	134168.9	134680.9	135937.3	10437.2
3PLM	133879.1	134647.1	136531.7	10523.8

None. 2PLM = Two Parameters Logistic Model, 3PLM = Three Parameters Logistic Model, -2LL = -2Log-Likelihood, AIC = Akaike's information criterion, BIC = Bayesian information criterion

Table S5 Item parameters for 121-item bank with 2PLM

**Item**	**Content**	***a***	***b***	**Item**	**Content**	***a***	***b***
1	Preventive medicine	0.512	0.021	62	Pathology	0.954	-0.171
2	Medicopsychology	0.852	0.971	63	Obstetrics and gynecology	0.886	-0.720
3	Immunology	0.872	-0.884	64	Obstetrics and gynecology	0.919	-1.267
4	Surgery	0.916	0.518	65	Surgery	0.985	-1.684
5	Microbiology	0.764	-0.191	66	Pathology	1.236	0.172

6	Internal medicine	0.834	-0.130	67	Pharmacology	0.983	-0.960
7	Pathophysiology	0.679	-1.517	68	Internal medicine	0.770	-1.989
8	Preventive medicine	0.620	-2.465	69	Physiology	1.568	-1.022
9	Pathology	0.777	0.657	70	Physiology	0.975	-0.134
10	Pathology	0.818	0.008	71	Pediatrics	0.915	-2.773
11	Microbiology	1.102	-2.338	72	Internal medicine	0.887	1.106
12	Pharmacology	0.919	-1.007	73	Pharmacology	1.124	-1.612
13	Pathophysiology	1.198	-0.537	74	Immunology	1.339	-0.906
14	Pharmacology	0.862	0.110	75	Pathophysiology	1.207	-1.868
15	Pharmacology	1.374	-0.926	76	Pharmacology	0.517	2.324
16	Surgery	0.544	0.486	77	Pharmacology	0.941	-0.680
17	Physiology	1.011	-0.845	78	Pathophysiology	0.801	-0.650
18	Pathology	0.755	0.462	79	Pathophysiology	0.946	-0.710
19	Medical ethics	1.795	-1.215	80	Pharmacology	0.924	-1.915
20	Medical ethics	1.695	-1.557	81	Pharmacology	1.451	-1.456
21	Pathology	1.574	-0.443	82	Pharmacology	1.097	0.922
22	Obstetrics and gynecology	0.785	-0.888	83	Biochemistry	1.306	-2.050
23	Obstetrics and gynecology	0.665	0.596	84	Pediatrics	1.292	2.133
24	Internal medicine	1.169	-2.286	85	Surgery	1.021	-2.068
25	Internal medicine	0.883	-2.585	86	Internal medicine	0.623	-0.986
26	Internal medicine	0.992	2.146	87	Physiology	1.546	-1.258
27	Biochemistry	0.752	-1.050	88	Surgery	0.775	-0.612
28	Immunology	0.793	-1.190	89	Internal medicine	1.592	-0.268
29	Immunology	0.939	0.430	90	Pathology	1.018	-1.316
30	Surgery	0.839	-0.452	91	anatomy	0.673	-0.039
31	Internal medicine	0.976	-1.387	92	Internal medicine	0.873	-0.396
32	Immunology	1.191	1.344	93	anatomy	1.436	-1.533
33	Preventive medicine	0.599	-0.194	94	Pathology	0.599	-0.881
34	Pathology	0.856	-0.204	95	Pathology	1.245	-0.382
35	Internal medicine	0.928	-0.271	96	Internal medicine	0.817	0.048
36	Internal medicine	0.779	-1.119	97	Immunology	1.193	-0.955
37	Microbiology	0.941	-1.947	98	Internal medicine	1.245	-0.481
38	Microbiology	1.112	0.398	99	Physiology	1.545	-0.665
39	Microbiology	0.818	-0.460	100	Internal medicine	0.752	-0.938
40	Physiology	0.639	-1.130	101	Pediatrics	0.773	-0.225
41	Internal medicine	1.619	-1.380	102	Internal medicine	0.985	-2.059
42	Internal medicine	1.270	-1.568	103	Biochemistry	1.596	-2.055
43	Pathology	0.792	-0.741	104	Pediatrics	0.917	-1.288
44	Surgery	0.853	-0.793	105	Obstetrics and gynecology	0.943	-0.427
45	Internal medicine	1.051	0.712	106	Internal medicine	1.930	-1.163
46	Pathology	0.895	-0.811	107	Anatomy	0.847	-0.976
47	Internal medicine	1.120	0.118	108	Internal medicine	1.186	-0.459
48	Surgery	1.534	-2.032	109	Internal medicine	1.196	-0.958
49	Sanitary legislation	0.965	-1.895	110	Internal medicine	1.165	-1.445
50	Sanitary legislation	1.167	-1.030	111	Internal medicine	1.184	-0.985
51	Sanitary legislation	0.965	-0.100	112	Internal medicine	1.154	-0.426
52	Internal medicine	1.766	-1.725	113	Microbiology	1.623	-2.657
53	Surgery	1.516	-1.151	114	Pathophysiology	0.775	0.489
54	Pathology	0.757	0.609	115	Pharmacology	0.839	-0.838
55	Internal medicine	0.779	-0.996	116	Pharmacology	0.818	-0.824
56	Internal medicine	1.086	-0.044	117	Pathology	1.081	-1.252
57	Internal medicine	1.004	-1.012	118	Obstetrics and gynecology	1.184	-0.890
58	Surgery	0.981	-1.954	119	Obstetrics and gynecology	1.137	0.536
59	Pathology	1.164	-1.929	120	Surgery	0.716	-0.370
60	Internal medicine	0.942	-1.327	121	Biochemistry	0.818	-0.211
61	Internal medicine	1.314	-1.063				

None. *a* = discrimination parameter, *b* = difficulty parameter

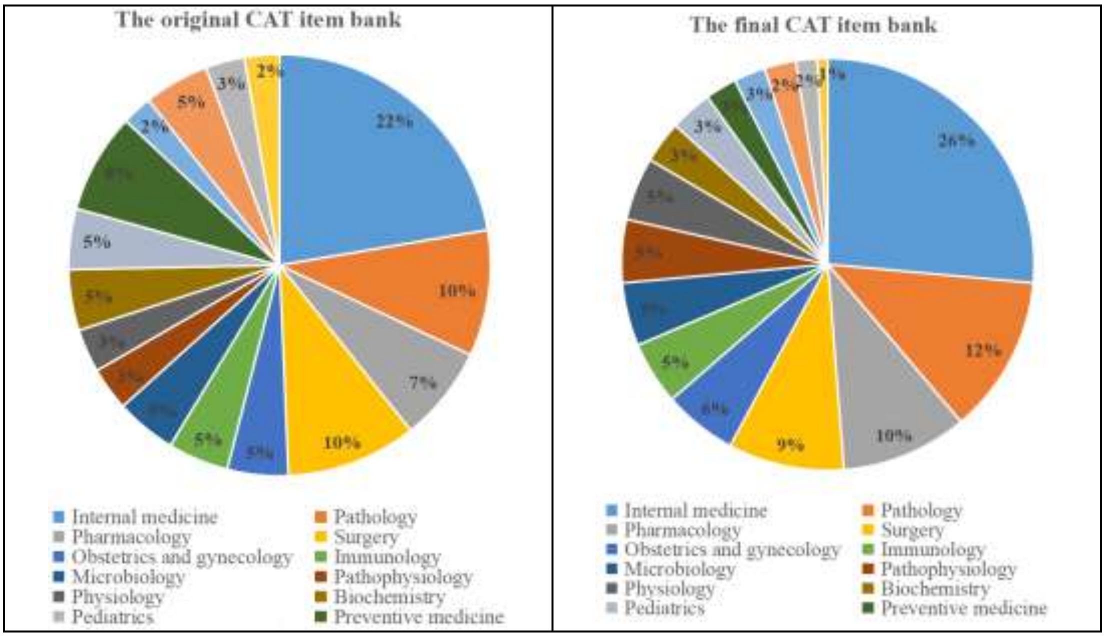

Fig. S1 Content distribution of the original and final item bank

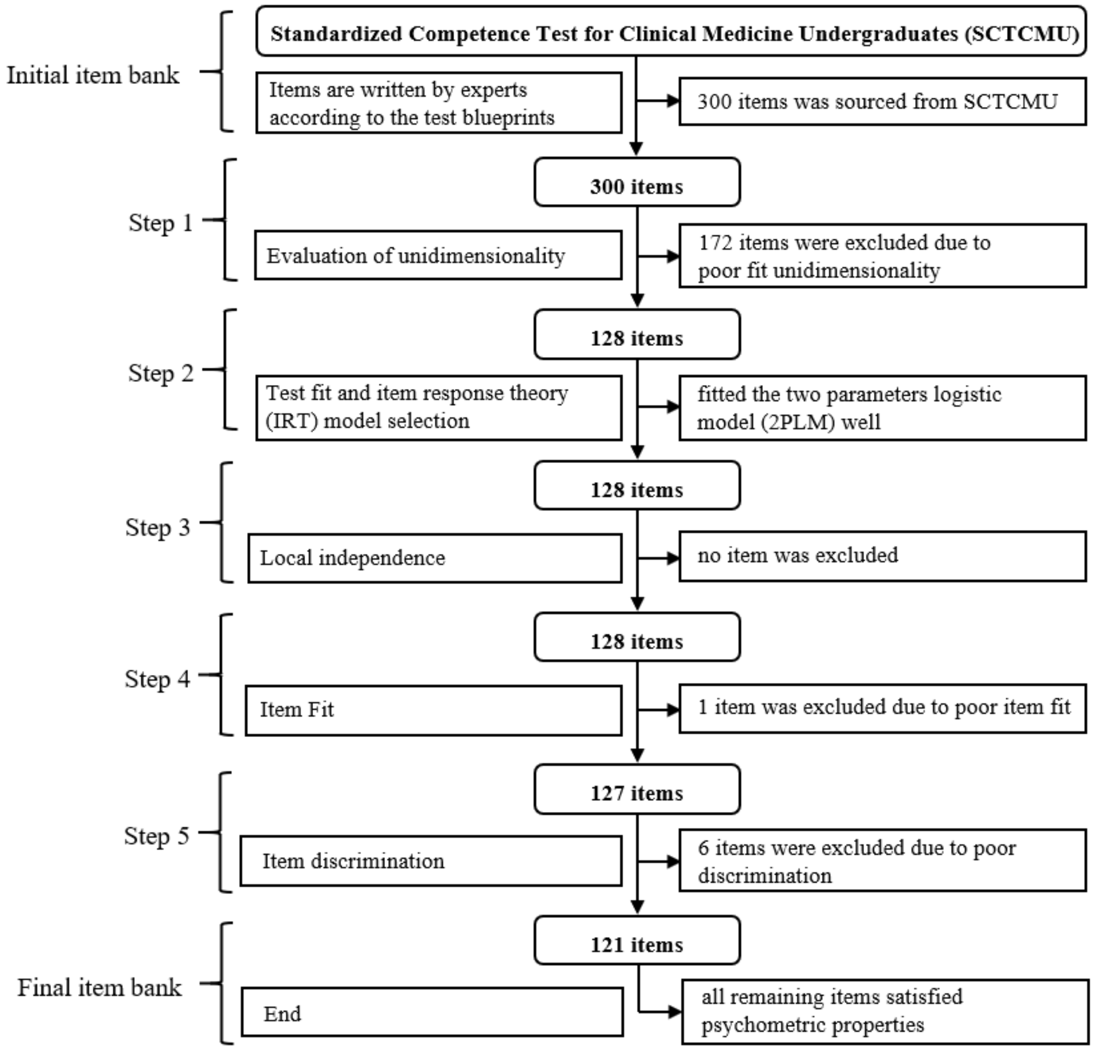

Fig. S2 Flowchart of the item bank development process

References

1. Reise SP, Morizot J, Hays RD. The role of the bifactor model in resolving dimensionality issues in health outcomes measures. Qual Life Res. 2007;16:19-31. doi: 10.1007/s11136-007-9183-7.
2. Kline RB. Principles and Practice of Structural Equation Modeling. New York: Guilford Press;2010.
3. Finch H, Habing B. Performance of DIMTEST-and NOHARM-based statistics for testing unidimensionality. Appl Psychol Meas. 2007;31:292-307. https://doi.org/10.1177/0146621606294490.
4. Maydeu-Olivares A, Joe H. Limited information goodness-of-fit testing in multidimensional contingency tables. Psychometrika. 2006;71:713.
5. Birnbaum A. On the estimation of mental ability. Series Rep. 1958;15:7755-7723.
6. Birnbaum AL. Some latent trait models and their use in inferring an examinee's ability. Statistical theories of mental test scores;1968.
7. Rasch G. Probabilistic models for some intelligence and attainment tests. Copenhagen: The Danish Institute of Educational Research. Chicago: The University of Chicago Press;1960.
8. Spiegelhalter DJ, Best NG, Carlin BP, Van der Linde A. Bayesian deviance, the effective number of parameters, and the comparison of arbitrarily complex models. Sci Rep. 1998;98-009.
9. Akaike H. A new look at the statistical model identification. IEEE Trans Automat Contr. 1974;19:716-723. doi: 10.1109/TAC.1974.1100705.
10. Schwarz G. Estimating the dimension of a model. Ann Stat. 1978;6:461-464.

11. Yen WM. Scaling performance assessments: Strategies for managing local item dependence. J Educ Meas. 1993;30:187-213. https://doi.org/10.1111/j.1745-3984.1993.tb00423.x
12. Flens G, Smits N, Terwee CB, Dekker J, Huijbrechts I, de Beurs E. Development of a computer adaptive test for depression based on the Dutch-Flemish version of the PROMIS item bank. Eval Health Prof. 2017;40:79-105. doi: 10.1177/0163278716684168
13. Köhler C, Hartig J. Practical significance of item misfit in educational assessments. Appl Psychol Meas. 2017;41:388-400. DOI: 10.1177/0146621617692978
14. Orlando M, Thissen D. Likelihood-based item-fit indices for dichotomous item response theory models. Appl Psychol Meas. 2000;24:50-64. https://doi.org/10.1177/01466216000241003
15. Orlando M, Thissen D. Further investigation of the performance of S-X2: An item fit index for use with dichotomous item response theory models. Appl Psychol Meas. 2003;27:289-298. https://doi.org/10.1177/0146621603027004004
16. Chang HH, Ying Z. A global information approach to computerized adaptive testing. Appl Psychol Meas. 1996;20:213-229. https://doi.org/10.1177/014662169602000303
17. Thundiyil JG, Modica RF, Silvestri S, Papa L. Do United States Medical Licensing Examination (USMLE) scores predict in-training test performance for emergency medicine residents? J Emerg Med. 2010;38(1):65-9. doi: 10.1016/j.jemermed.2008.04.010.

18. Tiffin PA, Illing J, Kasim AS, McLachlan JC. Annual Review of Competence Progression (ARCP) performance of doctors who passed Professional and Linguistic Assessments Board (PLAB) tests compared with UK medical graduates: national data linkage study. BMJ. 2014;348:g2622. doi: 10.1136/bmj.g2622.
19. Huh S. Application of computerized adaptive testing in medical education. Korean J Med Educ. 2009;21(2):97-102. DOI: 10.3946/kjme.2009.21.2.97.
20. De Champlain AF. A primer on classical test theory and item response theory for assessments in medical education. Med Educ. 2010 Jan;44(1):109-17. doi: 10.1111/j.1365-2923.2009.03425.x.
21. Downing SM. Item response theory: applications of modern test theory in medical education. Med Educ. 2003;37:739-45. doi: 10.1046/j.1365-2923.2003.01587.x. PMID: 12945568.
22. Han Y, Jiang Z, Ouyang J, Xu L, Cai T. Psychometric evaluation of a national exam for clinical undergraduates. Front Med (Lausanne). 2022;9:1037897. doi: 10.3389/fmed.2022.1037897.
23. Lahner FM, Schauber S, Lörwald AC, Kropf R, Guttormsen S, Fischer MR, Huwendiek S. Measurement precision at the cut score in medical multiple choice exams: Theory matters. Perspect Med Educ. 2020;9(4):220-228. doi: 10.1007/s40037-020-00586-0.
